# Health-related quality of life of children with attention-deficit/hyperactivity disorder versus children with diabetes and healthy controls

**DOI:** 10.1007/s00787-015-0728-y

**Published:** 2015-06-09

**Authors:** David Coghill, Paul Hodgkins

**Affiliations:** University of Dundee, Dundee, Scotland, UK; Shire, Wayne, PA USA; Division of Neuroscience, Medical Research Institute, Ninewells Hospital and Medical School, Dundee, DD1 9SY UK

**Keywords:** Attention deficit-hyperactivity disorder, Diabetes mellitus, Quality of life, Children

## Abstract

The impact of attention-deficit/hyperactivity disorder (ADHD) on health-related quality of life (HRQoL) is reported to be similar to that of other mental health and physical disorders. In this cross-sectional study, we hypothesized that children with ADHD and children with type 1 diabetes mellitus (T1DM) would have significantly worse HRQoL compared with healthy children, and that better clinical status in ADHD and T1DM would be associated with better HRQoL. Children were recruited from three outpatient services in Scotland. Responses to two frequently used validated HRQoL instruments, the Paediatric Quality of Life Inventory (PedsQL) and Child Health and Illness Profile-child edition (CHIP-CE), were obtained from parents/carers and children (6–16 years) with/without ADHD or T1DM. Child and parent/carer-completed HRQoL measurements were evaluated for 213 children with ADHD, 58 children with T1DM and 117 healthy children (control group). Significantly lower self and parent/carer ratings were observed across most PedsQL (*P* < 0.001) and CHIP-CE (*P* < 0.05) domains (indicating reduced HRQoL) for the ADHD group compared with the T1DM and control groups. Parent/carer and child ratings were significantly correlated for both measures of HRQoL (PedsQL total score: *P* < 0.001; CHIP-CE all domains: *P* < 0.001), but only with low-to-moderate strength. Correlation between ADHD severity and HRQoL was significant with both PedsQL and CHIP-CE for all parent/carer (*P* < 0.01) and most child (*P* < 0.05) ratings; more ADHD symptoms were associated with poorer HRQoL. These data demonstrate that ADHD has a significant impact on HRQoL (as observed in both parent/carer and child ratings), which seems to be greater than that for children with T1DM.

## Introduction

Health-related quality of life (HRQoL) is a complex, multifaceted construct that describes an individual’s perception of the impact of their health status on their physical, psychological and social functioning. HRQoL can be measured using a wide range of instruments [1–3]. Most of the currently available HRQoL measures are generic (non-disease specific). Generic measures used in clinical research include, for example, the Child Health Questionnaire Parent Form 50, Child Health and Illness Profile-child edition (CHIP-CE), Paediatric Quality of Life Inventory (PedsQL) and EuroQoL Five-Dimension Questionnaire [3, 4]. Generic HRQoL instruments assess broad constructs of HRQoL and can be used across different patients irrespective of their illness(es), but may not be sensitive to some disease-specific issues [3]. Disorder-specific instruments for attention-deficit/hyperactivity disorder (ADHD) include the ADHD Impact Module for children and adults, and the adult ADHD quality-of-life scale [5–7].

Clinicians, academics and regulators are becoming increasingly interested in the potential for HRQoL measures to be used as an index of clinical change and the effectiveness of therapeutic interventions [8, 9]. For example, HRQoL measures can play a role in identifying whether a particular treatment improves not only symptoms but also broader functioning, or whether treatments impact differentially on specific HRQoL domains. This information can lead to opportunities for the tailoring of treatments to address particular functional domains at either the individual or group level. Furthermore, regulatory authorities and reimbursement agencies believe indices of HRQoL can play an important role in their decision-making processes [9]. However, it is still the case that varying interpretations of the general construct and specific HRQoL domains, limited information concerning the agreement between self and proxy raters, lack of consensus with respect to the best HRQoL instruments to use, and lack of head-to-head comparative studies using psychometrically validated HRQoL instruments limit the usefulness of current measures [10]. Further validation of HRQoL measures to determine appropriate indices of reliable and clinically significant changes following therapy is required [11].

Attention-deficit/hyperactivity disorder is the most common behavioural childhood disorder and has a global prevalence in children of approximately 3.4–7.1 % [12–15]. It is characterized by developmentally inappropriate levels of hyperactivity/impulsivity and/or inattention, which impact many aspects of daily life and have a substantial burden on family functioning [16–18]. QoL in individuals with ADHD is impaired compared to that in individuals without the disorder [2, 3, 19, 20]. While the overall impact of ADHD on HRQoL has been reported to be comparable with other mental health conditions and physical disorders, including diabetes [3, 21–23], disparity still exists regarding the perceived impact of psychiatric and physical disorders, which can adversely affect aspects of mental health care provision such as funding allocation within health services [24]. ADHD also impacts negatively on the HRQoL of the parents/carers of those with the condition [25]. Unfortunately, most studies to date have only used parent/carer ratings [3], and our understanding of the child/adolescent’s views of their own HRQoL is limited. There are also only limited data on the association between clinical status and HRQoL.

To help address these issues, we conducted a cross-sectional investigation into the HRQoL of children with ADHD, a symptomatic (psychiatric) disorder, compared with that of children with type 1 diabetes mellitus (T1DM) and healthy controls. The ADHD and diabetic clinics from which patients were recruited for this study were responsible for all cases of these disorders within the local population and, therefore, had a broad range of presentations and clinical status, representative of local clinical practice. Both ADHD and T1DM are chronic disorders for which daily treatment is often required. Daily requirements for T1DM, such as the regular monitoring of blood sugar, balancing of insulin doses and managing of injections, are often difficult for parents/carers and children alike.

Findings on HRQoL in T1DM vary. While several studies have reported reduced HRQoL and particular disease-specific HRQoL issues, others have not [26–28]. Since starting our study, another research group has evaluated HRQoL in children aged 5–18 years with ADHD and comorbid psychiatric disorders compared with children with T1DM using patient- and parent-rated versions of the PedsQL [22]. In contrast to the current study, Limbers et al. focussed on the use of the PedsQL and did not evaluate HRQoL using any other instruments. The authors reported significantly poorer HRQoL in children with ADHD and comorbid psychiatric disorders compared with children with T1DM, supporting our inclusion of children with T1DM as a comparator in the current study.

Here, we report responses from children (6–16 years) with ADHD, T1DM or healthy controls, and their parents/carers, using two very different but commonly used validated generic paediatric HRQoL instruments: the PedsQL and the CHIP-CE questionnaires [29–32]. Our primary question was whether ADHD and T1DM impact on HRQoL, as measured by child and parent/carer ratings from these two different instruments. We also investigated the relationship between HRQoL and disease severity among the children with ADHD and T1DM. We hypothesized that compared with healthy children, children with ADHD and T1DM will both have significantly worse HRQoL, as rated by both self and parents/carers on both the PedsQL and CHIP-CE, and that better clinical status in ADHD and T1DM would be associated with better HRQoL.

## Methods

### Study design

Boys and girls aged 6–16 years were recruited from three outpatient services in Dundee, Scotland (ADHD, diabetic and dental). The dental clinics provide general dental services to the same local population as the ADHD and diabetic clinics; those invited to participate in this study were attending for a general check-up rather than specific treatments or emergency care. Postal invitations with an explanation and outline of study purpose were sent to all parents/carers of children attending the clinics via their respective clinical service or dentist (control group) prior to booked clinic appointments. If both the parent/carer and child could not participate, recruitment of either the parent/carer or child was acceptable.

The ADHD group consisted of children diagnosed with ADHD by an experienced child and adolescent psychiatrist trained in research methods, following a structured clinical assessment and using Diagnostic and Statistical Manual of Mental Disorders-IV criteria. Children were consecutive attendees at an ADHD continuing care clinic within the Tayside child and adolescent mental health service (CAMHS) at the Centre for Child Health, Dundee between September 2008 and January 2011. All children were receiving routine clinical care from the CAMHS service, which could include psychosocial and/or pharmacological treatment, or no treatment, for ADHD at the time of the study. The T1DM group consisted of children with T1DM (diagnosed subsequent to a fasting plasma glucose >7.8 mmol/L or random plasma glucose >11.1 mmol/L on two occasions; or glucose tolerance test: fasting plasma glucose >7.8 mmol/L and plasma glucose >11.1 mmol/L 2 h post 75 g oral glucose) who were attending a diabetic clinic at Ninewells Hospital, Dundee between September 2010 and July 2011 as part of their routine clinical care. The community control group consisted of children/adolescents attending the Lochee Dental Practice, Kings Cross Hospital or Dundee Dental Hospital between January 2012 and January 2013.

In the ADHD group, children were excluded if they had a history or current clinical diagnosis of other mental health disorders (i.e. bipolar disorder, cyclothymia, psychosis, major depressive disorder, eating disorder, clinically significant anxiety disorder or post-traumatic stress disorder, pervasive developmental disorder/autistic spectrum disorder), or who had moderate or severe intellectual disability (IQ < 50), or T1DM or any chronic medical condition requiring specialist treatment or requiring hospital admission for >24 h within the past year that may impact on HRQoL. Children with comorbid psychiatric disorders such as oppositional defiant disorder and conduct disorder were included in the study to increase the representativeness of the sample; however, these conditions must have been judged by the clinical team to be secondary to ADHD and not currently requiring treatment. In the T1DM group, children with a history or current diagnosis of mental health disorders (as above) or ADHD were excluded. In the control group, exclusion criteria were the same as for the ADHD and T1DM groups. Respondents who were considered unable to understand or complete questionnaires were also excluded from all study groups. No compensation was received for participating in the study.

Sample size for the primary outcome was calculated using data from previous HRQoL studies in ADHD [21, 33, 34]. At least 20 children per group were required to detect an effect size of at least 0.4 between the ADHD and control groups with an α error level of 5 % and a β error level of 50 % [35]. However, as we were also interested in comparisons between parent/carer and child outcomes, and between clinical and HRQoL outcomes, we elected to recruit a larger convenience sample.

### Data collection

The assessment procedure was the same for all groups, with questionnaire instructions provided in a standardized format and according to questionnaire-specific recommendations. The parent/carer and child completed questionnaires independently. A research assistant was available to ensure that the content and study procedures were understood and to check for completeness/missing data.

Two generic measures of HRQoL were used: 1) the child-self report and parent-proxy report versions of the PedsQL, which comprises four domains (physical, emotional, social functioning and school functioning) and a total score; and 2) the longer child-report and parent-report versions of the CHIP-CE, which comprises five domains (satisfaction, comfort, resilience, risk avoidance and achievement) but no total score. Both scales have been shown to be reliable and valid [29–32]. For both instruments, higher scores indicate better HRQoL.

Demographic data and medication status, as appropriate, were collected. Data on disease severity were collected for the ADHD group using the ADHD Rating Scale-IV (ADHD-RS-IV), completed by the clinician following interview with the parent/carer, and for the T1DM group using percentage glycated haemoglobin (HbA1_C_) [36, 37]. Socioeconomic status (SES) was measured using the Scottish Index of Multiple Deprivation (SIMD) [38] rank score. The SIMD ranks the 6505 geographical data zones that cover Scotland from most deprived (ranked 1) to least deprived (ranked 6505).

### Statistical analysis

All analyses were conducted using SPSS for Windows (v.21, SPSS Inc., Chicago, USA). Missing data were handled as per the scoring instructions for each instrument. Descriptive analyses [mean and standard deviation (SD)] were conducted for demographics and clinical variables. The scores for each HRQoL measurement completed by parents/carers and children with ADHD were compared with the responses from those with T1DM and healthy controls. All HRQoL and age data met assumptions of normality and homogeneity of variance group differences, and were analysed using multivariate analysis of covariance and thereafter by determination of simple effects through planned contrasts [39]. Correlations between child- and parent/carer-rated HRQoL, and between disorder severity and HRQoL, were tested using two-tailed bivariate correlations and reported using Pearson’s correlation coefficient. Statistical significance was set at *P* < 0.05.

## Results

Overall, 484 invitations were distributed (263 in the ADHD group, 74 in the T1DM group and 147 in the control group). In total, 388 children and parents/carers agreed to participate (80 %). All children and/or their parents/carers completed HRQoL measurements: 213 in the ADHD group, 58 in the T1DM group and 117 in the control group. There were significant differences between the groups with respect to age, sex and SES (Table 1). Children in the control group were younger than those in the ADHD and T1DM groups (mean age 10.1 vs 12.2 and 12.1 years, respectively) and there were more males in the ADHD group than in the T1DM and control groups (89.2 vs 43.1 % and 41.0 %, respectively). The T1DM group had a higher mean [SD] SIMD rank score (higher SES) than the ADHD and control groups (3239 [1954] vs 2449 [1796] and 2207 [1987], respectively); SES was similar in the ADHD and control groups.

**Table 1:** Child/adolescent demographics by study group

	ADHD group (*n* = 213)	T1DM group (*n* = 58)	Control group (*n* = 117)	*P* value
Mean (SD) age, years	12.2 (2.7)	12.1 (2.5)	10.1 (2.8)	<0.001*
Males, *n* (%)	190 (89.2)	25 (43.1)	48 (41.0)	<0.001**
Mean (SD) SIMD rank^a^	2449 (1796)	3239 (1954)	2207 (1987)	0.018***
Disease severity				
Mean ADHD rating scale total score (range; SD)	23.5 (1–50; 10.0)	NA	NA	–

*ADHD* attention-deficit/hyperactivity disorder, *NA* not available, *SD* standard deviation, *SIMD* Scottish Index of Multiple Deprivation, *T1DM* type 1 diabetes mellitus

* One-way analysis of variance *F* test (degrees of freedom: 2, 385) = 25.5

** Pearson *χ* ^2^ (2) = 98.2, *n* = 387

*** One-way analysis of variance *F* test (degrees of freedom: 2, 385) = 4.1

^a^Measure of socioeconomic status

As would be expected for a group of children in routine clinical care, there was a broad spectrum of symptom severity in the ADHD group. ADHD rating scale scores were normally distributed (Table 1). Age was included as a covariate in the analyses as it was significantly correlated with HRQoL domains on the PedsQL parent (school functioning, total score) and child (school functioning) scales, and the CHIP-CE parent (satisfaction, resilience, risk avoidance, achievement) and child (all five domains) scales. In all cases, increasing age was associated with lower HRQoL, except for the comfort domain on the child-rated CHIP-CE scale, for which increasing age was associated with improved HRQoL. SIMD rank was also included as a covariate owing to correlations between SIMD rank scores and PedsQL parent (social and school functioning, total score) and child (school functioning) scales, and the CHIP-CE parent (risk avoidance) and child (comfort) ratings. Lower SES was associated with a lower HRQoL. There were no significant differences in any domain of any scale for any of the groups with respect to sex; therefore, sex was not included as a covariate in the analyses. The HRQoL scores for the control group were consistent with population norms [30, 31, 40].

### Between-group comparisons

Compared with the T1DM and control groups, children with ADHD had significantly lower PedsQL ratings (indicating poorer HRQoL) across all domains for both self and parent/carer ratings (*P* < 0.001; Tables 2, 3). For the CHIP-CE, there were again significantly lower ratings (*P* < 0.05 to <0.001) for the ADHD group compared with both control and T1DM groups for all parent/carer-rated domains and for child ratings on the comfort, risk avoidance and achievement domains (Tables 4, 5). Mean scores were similar for the T1DM and control groups for both the parent/carer-rated and the child-rated HRQoL domains of both instruments.

**Table 2:** PedsQL parent/carer scores by study group

Domain	Study group^a^	Unadjusted mean score (SD)	Adjusted mean score^b^ (SE)	95 % confidence interval^c^	MANCOVA				Effect size (δ)	
					*F* test	*df*	*P* value		ADHD vs Control	ADHD vs T1DM
Physical functioning	ADHD	73.7 (18.6)	73.4 (1.4)	70.7, 76.2	24.2	2, 327	<0.001	ADHD < Control, T1DM	0.96	0.90
	T1DM	87.8 (12.9)	87.0 (3.0)	81.1, 92.9						
	Control	89.3 (14.1)	90.1 (2.1)	86.0, 94.1						
Emotional functioning	ADHD	53.4 (22.2)	53.0 (1.7)	49.7, 56.3	53.6	2, 327	<0.001	ADHD < Control, T1DM	1.46	1.27
	T1DM	77.3 (16.0)	76.3 (3.6)	69.3, 83.4						
	Control	81.6 (17.1)	82.9 (2.5)	78.0, 87.8						
Social functioning	ADHD	62.9 (25.5)	62.5 (1.8)	58.9, 66.1	45.0	2, 327	<0.001	ADHD < Control, T1DM	1.38	1.18
	T1DM	87.5 (16.6)	85.8 (3.9)	78.2, 93.4						
	Control	90.2 (14.5)	91.8 (2.7)	86.5, 97.0						
School functioning	ADHD	52.2 (21.2)	52.0 (1.5)	49.0, 55.1	92.9	2, 327	<0.001	ADHD < T1DM < Control^d^	2.08	1.50
	T1DM	79.5 (16.0)	78.0 (3.2)	71.5, 84.4						
	Control	87.7 (14.1)	88.6 (2.3)	84.2, 93.1						
Total score	ADHD	62.1 (16.5)	61.8 (1.2)	59.4, 64.2	78.8	2, 327	<0.001	ADHD < Control, T1DM	1.61	1.51
	T1DM	83.8 (12.5)	82.6 (2.6)	77.5, 87.8						
	Control	87.2 (11.8)	88.2 (1.8)	84.6, 91.8						

*ADHD* attention-deficit/hyperactivity disorder, *df* degrees of freedom, *MANCOVA* multivariate analysis of covariance, *PedsQL* Paediatric Quality of Life Inventory, *SD* standard deviation, *SE* standard error, *T1DM* type 1 diabetes mellitus

^a^ *n* = 206 in ADHD group, *n* = 54 in T1DM group and *n* = 110 in control group

^b^Adjusted for age and socioeconomic status

^c^For adjusted mean score

^d^Effect size (δ): T1DM vs control (School functioning) = 0.55

**Table 3:** PedsQL child scores by study group

Domain	Study group^a^	Unadjusted mean score (SD)	Adjusted mean score^b^ (SE)	95 % confidence interval^c^	MANCOVA				Effect size (δ)	
					*F* test	*df*	*P* value		ADHD vs Control	ADHD vs T1DM
Physical functioning	ADHD	79.0 (15.7)	78.3 (1.2)	76.0, 80.7	14.2	2, 321	<0.001	ADHD < Control, T1DM	0.57	0.60
	T1DM	87.4 (12.2)	86.8 (2.5)	81.8, 91.7						
	Control	87.7 (14.9)	89.3 (1.8)	85.8, 92.7						
Emotional functioning	ADHD	69.5 (21.7)	68.9 (1.7)	65.6, 72.2	14.9	2, 321	<0.001	ADHD < Control, T1DM	0.75	0.45
	T1DM	78.7 (19.2)	77.4 (3.4)	70.7, 84.2						
	Control	83.5 (15.6)	85.2 (2.4)	80.5, 90.0						
Social functioning	ADHD	76.0 (22.5)	75.0 (1.7)	71.6, 78.3	14.8	2, 321	<0.001	ADHD < Control, T1DM	0.62	0.59
	T1DM	86.9 (14.3)	85.3 (3.5)	78.4, 92.2						
	Control	88.5 (18.0)	91.2 (2.5)	86.3, 96.0						
School functioning	ADHD	60.8 (21.8)	61.0 (1.7)	57.7, 64.4	32.8	2, 321	<0.001	ADHD < T1DM < Control^d^	1.37	0.61
	T1DM	73.7 (20.8)	73.5 (3.5)	66.6, 80.4						
	Control	86.3 (16.0)	86.0 (2.5)	81.1, 90.9						
Total score	ADHD	72.1 (15.5)	71.5 (1.2)	69.2, 73.9	30.7	2, 321	<0.001	ADHD < T1DM < Control^d^	1.07	0.72
	T1DM	82.2 (12.4)	81.3 (2.4)	76.5, 86.1						
	Control	86.6 (11.8)	88.0 (1.7)	84.6, 91.4						

*ADHD* attention-deficit/hyperactivity disorder, *df* degrees of freedom, *MANCOVA* multivariate analysis of covariance, *PedsQL* Paediatric Quality of Life Inventory, *SD* standard deviation, *SE* standard error, *T1DM* type 1 diabetes mellitus

^a^ *n* = 174 in ADHD group, *n* = 45 in T1DM group and *n* = 106 in control group

^b^Adjusted for age and socioeconomic status

^c^For adjusted mean score

^d^Effect size (δ): T1DM vs control (School functioning) = 0.69, T1DM vs control (total score) = 0.36

**Table 4:** CHIP-CE parent/carer scores by study group

Domain	Study group^a^	Unadjusted mean score (SD)	Adjusted mean score^b^ (SE)	95 % confidence interval^c^	MANCOVA				Effect size (δ)	
					*F* test	*df*	*P* value		ADHD vs Control	ADHD vs T1DM
Satisfaction	ADHD	32.9 (16.3)	34.2 (1.3)	31.6, 36.8	8.7	2, 311	<0.001	ADHD < Control, T1DM	0.68	0.81
	T1DM	44.1 (11.5)	45.5 (2.9)	39.8, 51.2						
	Control	44.1 (16.7)	41.1 (1.9)	37.3, 44.8						
Comfort	ADHD	42.5 (13.5)	42.4 (1.0)	40.4, 44.4	19.9	2, 311	<0.001	ADHD < Control, T1DM	0.93	0.76
	T1DM	51.3 (9.7)	51.0 (2.3)	46.4, 55.5						
	Control	53.5 (10.3)	53.7 (1.5)	50.7, 56.7						
Resilience	ADHD	37.7 (14.7)	38.9 (1.1)	36.7, 41.0	3.8	2, 311	0.022	ADHD < Control, T1DM	0.62	0.46
	T1DM	43.6 (11.2)	44.7 (2.4)	40.0, 49.4						
	Control	45.7 (11.0)	43.0 (1.6)	39.9, 46.1						
Risk avoidance	ADHD	28.7 (15.5)	28.5 (1.1)	26.3, 30.6	79.0	2, 311	<0.001	ADHD < Control, T1DM	1.88	1.84
	T1DM	49.7 (8.2)	48.8 (2.4)	44.0, 53.6						
	Control	50.7 (8.9)	51.5 (1.6)	48.3, 54.7						
Achievement	ADHD	31.8 (12.0)	32.2 (0.9)	30.5, 34.0	73.5	2, 311	<0.001	ADHD < Control, T1DM	1.96	1.57
	T1DM	48.7 (9.9)	48.7 (2.0)	44.8, 52.5						
	Control	50.7 (7.8)	50.0 (1.3)	47.3, 52.5						

*ADHD* attention-deficit/hyperactivity disorder, *CHIP*-*CE* Child Health and Illness Profile–child edition, *df* degrees of freedom, *MANCOVA* multivariate analysis of covariance, *SD* standard deviation, *SE* standard error, *T1DM* type 1 diabetes mellitus

^a^ *n* = 173 in ADHD group, *n* = 42 in T1DM group and *n* = 100 in control group

^b^Adjusted for age and socioeconomic status

^c^For adjusted mean score

**Table 5:** CHIP-CE child scores by study group

Domain	Study group^a^	Unadjusted mean score (SD)	Adjusted mean score^b^ (SE)	95 % confidence interval^c^	MANCOVA				Effect size (δ)	
					*F* test	*df*	*P* value		ADHD vs Control	ADHD vs T1DM
Satisfaction	ADHD	44.5 (11.1)	45.1 (0.9)	43.3, 47.0	0.5	2, 328	NS	ADHD = Control = T1DM	NS	NS
	T1DM	43.7 (10.3)	44.8 (1.9)	41.0, 48.5						
	Control	45.2 (12.0)	43.4 (1.4)	40.8, 46.1						
Comfort	ADHD	53.5 (8.6)	53.0 (0.7)	51.7, 54.3	5.0	2, 328	0.008	ADHD < Control, T1DM	0.17	0.51
	T1DM	57.1 (5.1)	56.2 (1.4)	53.5, 58.9						
	Control	54.9 (7.8)	56.3 (1.0)	54.4, 58.1						
Resilience	ADHD	43.9 (10.8)	44.3 (0.8)	42.6, 45.9	2.3	2, 328	NS	ADHD = Control = T1DM	NS	NS
	T1DM	47.2 (9.7)	47.8 (1.7)	44.3, 51.1						
	Control	47.4 (7.9)	46.5 (1.2)	44.1, 48.9						
Risk avoidance	ADHD	39.8 (10.7)	40.4 (0.8)	38.9, 41.9	30.1	2, 328	<0.001	ADHD < Control, T1DM	1.47	0.76
	T1DM	47.4 (9.3)	47.9 (1.6)	44.8, 51.0						
	Control	51.9 (5.7)	50.4 (1.1)	48.2, 52.6						
Achievement	ADHD	39.4 (11.3)	39.7 (0.9)	38.0, 41.5	10.0	2, 328	<0.001	ADHD < Control, T1DM	0.25	0.72
	T1DM	46.7 (9.0)	47.0 (1.8)	43.5, 50.6						
	Control	42.2 (10.9)	45.0 (1.3)	42.5, 47.5						

*ADHD* attention-deficit/hyperactivity disorder, *CHIP*-*CE* Child Health and Illness Profile–child edition, *df* degrees of freedom, *MANCOVA* multivariate analysis of covariance, *NS* not significant, *SD* standard deviation, *SE* standard error, *T1DM* type 1 diabetes mellitus

^a^ *n* = 180 in ADHD group, *n* = 46 in T1DM group and *n* = 106 in control group

^b^Adjusted for age and socioeconomic status

^c^For adjusted mean score

### Association between parent/carer and child ratings

Parent/carer and child ratings were significantly correlated across the different domains for both measures of HRQoL, apart from the social functioning and school functioning domains of the PedsQL (Table 6). However, these correlations were only low to moderate in strength (PedsQL total score: *r* = 0.546, *P* < 0.001; CHIP-CE all domains: *r* > 0.274, *P* < 0.001). Domains demonstrating significant correlations largely remained significant when data were analysed separately by study group and by age for children aged 6 to <11 years and adolescents aged ≥11 years (Table 6).

**Table 6:** Correlations between parent/carer and child ratings of HRQoL measurements

PedsQL	Physical functioning	Emotional functioning	Social functioning	School functioning	Total score
All children (*n* = 335)	0.357***	0.439***	0.080	0.097	0.546***
ADHD (*n* = 199)	0.289***	0.365***	0.081	0.107	0.440***
T1DM (*n* = 51)	0.518***	0.395***	0.313*	0.500***	0.616***
Control (*n* = 117)	0.231*	0.280**	0.162	0.322**	0.290**
Age group					
6 to <11 years (*n* = 138)	0.279***	0.390***	0.385***	0.497***	0.453***
≥11 years (*n* = 222)	0.405***	0.475***	0.102	0.113	0.593***

CHIP-CE	Satisfaction	Comfort	Resilience	Risk avoidance	Achievement
All children (*n* = 339)	0.274***	0.302***	0.310***	0.647***	0.517***
ADHD (*n* = 205)	0.380***	0.325***	0.283***	0.534***	0.442***
T1DM (*n* = 54)	0.466**	0.491**	0.320*	0.648***	0.540***
Control (*n* = 116)	0.146	0.188*	0.205*	0.333***	0.417***
Age group					
6 to < 11 years (*n* = 142)	0.122	0.220*	0.188*	0.483***	0.413***
≥11 years (*n* = 231)	0.348***	0.394***	0.356***	0.719***	0.558***

*ADHD* attention-deficit/hyperactivity disorder, *CHIP*-*CE* Child Health and Illness Profile–child edition, *HRQoL* health-related quality of life, *PedsQL* Paediatric Quality of Life Inventory, *T1DM* type 1 diabetes mellitus

Pearson’s correlation: * P < 0.05, ** P < 0.01, *** P < 0.001

### Association between HRQoL and disease severity

For the ADHD group, disease severity (ADHD-RS-IV) and HRQoL were significantly correlated for all parent/carer ratings and for most child ratings (except PedsQL physical functioning and CHIP-CE satisfaction and resilience) on both HRQoL scales (Table 7). In all cases, more ADHD symptoms were associated with poorer HRQoL. Correlations were again in the low-to-moderate range and were higher for parent/carer ratings than for child ratings. For the T1DM group, the only significant correlations between disease severity (HbA1_C_) and HRQoL were for child-rated school functioning on the PedsQL scale (*r* = −0.343, *P* = 0.02), and child-rated satisfaction (*r* = −0.292, *P* = 0.03), resilience (*r* = −0.304, *P* = 0.02) and risk avoidance (*r* = −0.303, *P* = 0.03) on the CHIP-CE scale (Table 7). Where there were significant correlations, worse diabetic control was associated with poorer HRQoL.

**Table 7:** Correlations between parent/carer and child ratings of HRQoL and disease severity data

ADHD group (ADHD-RS-IV)					
PedsQL	Physical functioning	Emotional functioning	Social functioning	School functioning	Total score
Parent/carer ratings	−0.296**	−0.451***	−0.387***	−0.542***	−0.545***
Child ratings	−0.119	−0.302**	−0.244*	−0.292**	−0.277**

ADHD group (ADHD-RS-IV)					
CHIP-CE	Satisfaction	Comfort	Resilience	Risk avoidance	Achievement
Parent/carer ratings	−0.302***	−0.351**	−0.290**	−0.433***	−0.453***
Child ratings	−0.092	−0.306**	0.074	−0.354**	−0.236*

T1DM group (HbA1_C_)					
PedsQL	Physical functioning	Emotional functioning	Social functioning	School functioning	Total score
Parent/carer ratings	−0.256	−0.174	−0.166	−0.001	−0.144
Child ratings	−0.223	−0.118	0.074	−0.343*	−0.176

T1DM group (HbA1_C_)					
CHIP-CE	Satisfaction	Comfort	Resilience	Risk avoidance	Achievement
Parent/carer ratings	0.022	−0.173	−0.089	−0.051	−0.018
Child ratings	−0.292*	−0.250	−0.304*	−0.303*	−0.152

*ADHD* attention-deficit/hyperactivity disorder, *ADHD*-*RS*-*IV* ADHD Rating Scale-IV, *CHIP*-*CE* Child Health and Illness Profile–child edition, *HbA1*_*C*_ glycated haemoglobin, *HRQoL* health-related quality of life, *PedsQL* Paediatric Quality of Life Inventory, *T1DM* type 1 diabetes mellitus

Pearson’s correlation: * *P* < 0.05, ** *P* < 0.01, *** *P* < 0.001

## Discussion

This study provides child- and parent/carer-rated HRQoL data from two validated instruments for children with ADHD receiving routine clinical care, children with T1DM and healthy controls. Despite being on treatment, the reported HRQoL of children with ADHD was lower than those of the T1DM or control groups for all parent/carer ratings with both instruments, all child ratings with PedsQL and most child ratings with CHIP-CE. Results for PedsQL-measured HRQoL are consistent with previous findings from Limbers et al. in children with ADHD and comorbid psychiatric disorders compared with children with T1DM [22]. Limbers et al. specifically selected children with ADHD and comorbid psychiatric disorders, including mood, anxiety, psychotic and substance use disorders, learning disabilities/mental retardation and other psychiatric disorders, to reflect the high prevalence of comorbidities in patients attending a Pediatric Psychiatric Clinic. Children with mental health disorders that were not secondary to ADHD or which required treatment were excluded from the current study, suggesting that the negative impact of ADHD on HRQoL is measureable in the absence of cormorbid psychiatric disorders, such as mood, anxiety, psychotic and substance use disorders, which may themselves reduce HRQoL. It should be noted that children with ADHD and chronic medical conditions requiring specialist medical attention were also excluded from the current study. Evidence is emerging to suggest that children with ADHD may have an increased risk for certain medical conditions compared with children without the disorder, for example, obesity and asthma [41, 42]. It is likely that the burden of illness for children with ADHD may be greater than reported here.

The PedsQL and CHIP-CE are both modular, multi-domain, validated, generic HRQoL measures with child and proxy ratings. The findings were generally consistent for the two different instruments; further analyses are required to identify whether both instruments are measuring similar aspects of HRQoL or whether they are indexing similar levels of disruption across different aspects.

The effect sizes for parent/carer and child ratings were similar for both instruments, although the strength of effect was somewhat less for several of the child-completed CHIP-CE domains than was seen for the other measures. Parent and child ratings were significantly correlated with each other on both scales. However, the strength of both effect and correlation varied by domain, and child–parent/carer correlations did not reach significance for the social functioning and school functioning domains of the PedsQL. Our findings of a strong child-rated effect on the PedsQL and several of the CHIP-CE domains contrast with previous studies that reported reductions in parent but not child-rated HRQoL in ADHD [3, 22, 43–45]. This has often been interpreted as a tendency for children with ADHD to minimize their difficulties [3]. The finding that the child-rated PedsQL scores were more consistently reduced than those for the CHIP-CE may indicate that some of the previous findings may be instrument dependent. However, the lower child–parent/carer correlations on some PedsQL domains suggest a more complex relationship between the two versions of the two instruments. Interestingly, when these correlations were looked at by age group, the adolescent–parent/carer correlations tended to be stronger than those for child–parent/carer in this study, apart from the social functioning and school functioning PedsQL domains. One might have expected parents/carers to have been more in tune with the younger children than the adolescents who spend more time outside the home/with peers. However, the positive correlations between scores in parents/carers and children of all ages suggest that parents/carers are reasonably good at assessing the QoL of their children. While adolescents are also good at evaluating their own QoL, younger children may not grasp the concepts and constructs presented by the instruments and are thus less able to give an accurate account.

In contrast to the limited correlations between disease severity and HRQoL for T1DM, the correlations between disease severity and ADHD were significant on both scales for all parent/carer and most child ratings (although PedsQL seemed more sensitive for the child ratings than CHIP-CE). However, these correlations were only low to moderate in strength, suggesting that, although there is overlap between the two, they are assessing different aspects of functioning. Both disease severity and quality of care impact HRQoL and, in the present study, fewer symptoms were associated with better HRQoL. These findings clearly carry a significant message to those delivering clinical care to patients with ADHD: that optimizing symptomatic control will have a positive impact on HRQoL. This is important as most studies investigating quality of routine clinical care suggest that treatment outcomes are often not optimized [46] and that clinicians tend to be satisfied with identifying some improvement rather than striving to maximize symptom reduction [47]. The Multimodal Treatment of ADHD (MTA) study clearly demonstrated that closely managed treatment was superior to treatment in the community [48]. Even though these findings are mentioned in many clinical guidelines, there is currently little evidence that they have been implemented in routine clinical practice (although evidence-based clinical protocols are beginning to emerge) (Coghill D, Personal communication, 25 January 2014). Although ADHD symptoms and HRQoL were correlated in this study, it is still possible that the PedsQL and CHIP-CE scales are measuring different aspects of the problems faced by those with ADHD. Correlations between symptoms and HRQoL are far from perfect, suggesting that, even if symptoms are optimally treated, there are still likely to be other factors that need to be addressed to optimize HRQoL and clinical functioning.

The HRQoL of participants with T1DM in this study was not impaired compared with the control group. Although these findings were not expected at the outset of the study, they are in keeping with the results of a more recent systematic review [49]. Although the authors of this review concluded that T1DM does not impact negatively on HRQoL as measured by generic instruments (such as those used in the current study), they noted that disease-specific impairments in HRQoL are present in children with T1DM. This finding may reflect greater attention being paid to optimizing care in this patient group and/or a lower impact of T1DM on day-to-day functioning than ADHD. At the very least, these data support current attempts seeking parity in mental and physical health services [50].

The limitations of this cross-sectional HRQoL study include non-response to study invitations, which can result in bias, and as data on non-responders were unavailable, differences in the demographic and disease characteristics between responders and non-responders could not be evaluated. In addition, group sizes were not equally matched and there were age, sex and SES differences between groups. The bias towards males observed in the ADHD group is typical of clinical samples; however, sex did not appear to affect HRQoL, and age and SES were adjusted for in the analyses. While patients attending the ADHD and T1DM clinics were initially referred for treatment and, therefore, not an epidemiological sample, attendees were recruited sequentially from the whole clinic populations. Although patients in the ADHD group were receiving clinical care for ADHD, medication status was not collected and could not be included in the analyses. As parents/carers and children both contributed information to the clinician-scored ratings of ADHD symptoms (ADHD-RS-IV) and HRQoL, these ratings were not entirely independent of each other. This may have inflated the correlations between the two. However, while correlations between these measures were significant, they were not highly correlated, supporting the value of measuring HRQoL in addition to symptoms.

These data demonstrate that ADHD has a significant impact on generic HRQoL, and that this appears to be greater than seen for T1DM. This impact also appears to be related to ADHD severity and, importantly, is observed in both parent/carer and child ratings. We consider that these findings should be generalizable, at least for a referred population of patients. Furthermore, the degree of impairment of HRQoL reported here is similar to that seen in other studies, and agreement was observed between the parents/carers and children, and between the two measures. However, the findings are preliminary and it may be prudent to obtain ratings from both instruments for an accurate scientific perspective of HRQoL. We believe that further studies are necessary to obtain a consensus opinion of the validity and value of HRQoL measures.
